# Diversity of Maize Shoot Apical Meristem Architecture and Its Relationship to Plant Morphology

**DOI:** 10.1534/g3.115.017541

**Published:** 2015-03-05

**Authors:** Addie M. Thompson, Jianming Yu, Marja C. P. Timmermans, Patrick Schnable, James E. Crants, Michael J. Scanlon, Gary J. Muehlbauer

**Affiliations:** * Department of Agronomy and Plant Genetics, University of Minnesota, St. Paul, Minnesota 55108; † Cold Spring Harbor Laboratory, Cold Spring Harbor, New York 11724; ‡ Department of Plant Biology, Cornell University, Ithaca, New York 14853; § Department of Agronomy, Iowa State University, Ames, Iowa 50011; ** Department of Plant Biology, University of Minnesota, St. Paul, Minnesota 55108

**Keywords:** shoot apical meristem, QTL, maize development, NAM, heterosis

## Abstract

The shoot apical meristem contains a pool of undifferentiated stem cells and controls initiation of all aerial plant organs. In maize (*Zea mays*), leaves are formed throughout vegetative development; on transition to floral development, the shoot meristem forms the tassel. Due to the regulated balance between stem cell maintenance and organogenesis, the structure and morphology of the shoot meristem are constrained during vegetative development. Previous work identified loci controlling meristem architecture in a recombinant inbred line population. The study presented here expanded on this by investigating shoot apical meristem morphology across a diverse set of maize inbred lines. Crosses of these lines to common parents showed varying phenotypic expression in the F1, with some form of heterosis occasionally observed. An investigation of meristematic growth throughout vegetative development in diverse lines linked the timing of reproductive transition to flowering time. Phenotypic correlations of meristem morphology with adult plant traits showed an association between the meristem and flowering time, leaf shape, and yield traits, revealing links between the control and architecture of undifferentiated and differentiated plant organs. Finally, quantitative trait loci mapping was utilized to map the genetic architecture of these meristem traits in two divergent populations. Control of meristem architecture was mainly population-specific, with 15 total unique loci mapped across the two populations with only one locus identified in both populations.

The shoot apical meristem (SAM) produces all of the plant’s above-ground organs and performs the dual functions of organogenesis and stem cell maintenance. A balance of these two roles is maintained to prevent stem cell pool depletion and overproliferation, which lead to developmental arrest or abnormal leaf formation, respectively (Barton 2010). In maize, 100–200 founder cells act as leaf initials (Poethig 1984), and organogenesis takes place in the peripheral zone of the SAM. Between fertilization and seed maturation/quiescence, approximately five leaves are formed in the typical maize embryo. On germination, leaf initiation resumes at regular intervals, referred to as plastochrons (P) (Sharman 1942). Vegetative growth of the SAM occurs in three stages: an initial growth phase, size maintenance with little growth, and another period of rapid growth immediately prior to transition into an inflorescence shoot meristem (Thompson *et al.* 2014). Studies of SAM function have revealed many regulatory processes affecting growth and development; these investigations primarily have been analysis of developmental mutants, many of which alter whole plant and/or SAM morphology.

Multiple and redundant genetic mechanisms contribute to SAM initiation, growth, and function. These include the CLAVATA/WUSCHEL pathway (Taguchi-Shiobara *et al.* 2001; Bommert *et al.* 2013) and the *Knotted-1-like homeobox (KNOX)* genes (Hake *et al.* 1995; Kerstetter *et al.* 1994; Jackson *et al.* 1994), both of which impact SAM size in maize (Nishimura *et al.* 2000, Rosin *et al.* 2003). *Knotted1-E1* (*kn1-E1*) loss-of-function mutants lack meristem maintenance (Kerstetter *et al.* 1997) and show a range of penetrance of SAM size phenotypes across different inbred backgrounds (Vollbrecht *et al.* 2000). Plant hormones (including auxin, cytokinin, gibberellins, and brassinosteroids) (see reviews in Hay *et al.* 2004 and Vanstraelen and Benková 2012) and chromatin remodeling factors (Efroni *et al.* 2013; Shen and Xu 2009) also contribute to maintaining the balance between stem cell maintenance and organogenesis in the SAM. Other important regulatory pathways in the SAM involve small RNAs (Zhang *et al.* 2006; microRNA review in Axtell 2013) and *trans*-acting small interfering RNAs pathways (Nogueira *et al.* 2007; Douglas *et al.* 2010), as well as downstream factors involving changes in cell wall properties and metabolic processes (Kierskowski *et al.* 2012; Peaucelle *et al.* 2011; Woodward *et al.* 2010). Mutations in these pathways also impact whole plant phenotypes, as seen in the maize mutants *fasciated ear2* (Taguchi-Shiobara *et al.* 2001), *compact plant2* (Bommert *et al.* 2013), and *bladekiller1* (Woodward *et al.* 2010), among many others.

The relationship between undifferentiated tissues and differentiated tissues is relatively unexplored. Several groups have focused on determining the genetic control of plant architecture via quantitative trait locus (QTL) mapping experiments. Previous studies of maize morphology have identified QTL for shoot architecture (Lauter *et al.* 2008), leaf shape (Tian *et al.* 2011), root architecture (Hochholdinger and Tuberosa 2009), inflorescence architecture (Upadyayula *et al.* 2006; Brown *et al.* 2011), and flowering time (Buckler *et al.* 2009). There have been fewer investigations, however, into the architecture of undifferentiated plant structures such as the SAM (Thompson *et al.* 2014). Describing the relationship of the architecture and genetic control of undifferentiated structures such as the SAM and those of differentiated plant parts, such as leaf morphology, plant height, flowering time, and inflorescence architecture, may lead to important insights into plant development and the regulators of differentiated plant structure morphology.

A previous investigation suggested that much of the control of the natural variation present in SAM architecture takes place outside of known major meristem regulators, as evidenced by a lack of overlap with genes known to cause mutant phenotypes in the SAM (Thompson *et al.* 2014). This study focused on one population (IBMRIL) and did not encompass a wide diversity of maize genotypes. Larger-effect genes contributing to meristem morphology may not be segregating in this particular population, and the range of diversity present for SAM architecture across a wider variety of maize backgrounds is unknown. Furthermore, the timeline of SAM growth across vegetative development may vary in more highly divergent inbred lines. Two other unexplored areas of maize meristem architecture are the extent of heterosis present for SAM traits and the relationship of these traits to adult plant morphology.

The objectives of this study were to: survey maize SAM architecture in a panel of diverse inbred lines; test for the presence and extent of heterosis in crosses made among diverse lines; investigate SAM growth throughout vegetative development in genotypes with contrasting morphologies, backgrounds, and flowering times; characterize phenotypic correlations between undifferentiated and differentiated plant structures (connecting maize SAM architecture to adult plant morphology); and map QTL for SAM morphology in two RIL populations created from highly divergent parents to determine the extent of shared genetic control in different backgrounds.

## Materials and Methods

### Plant materials

This study utilized the 27 nested association mapping (NAM) founder inbreds (includes Mo17 and B73) as well as individuals from two RIL subpopulations (CML277 and P39) of the NAM (Supporting Information, Table S1) (Yu *et al.* 2008). The intermated B73 × Mo17 recombinant inbred line (IBM RIL) population was also used (Lee *et al.* 2002), as well as F1 offspring of eight inbreds (B97, Hp301, IL14H, Ms71, NC358, Oh43, Oh7B, and P39) crossed to B73 and Mo17. Eighteen diverse inbreds (Table S1) selected to represent a wide range of flowering times were utilized in the time course experiment.

### Plant growth and experimental design

The NAM founders, the two NAM RIL subpopulations, and the B73 × NAM founder F1 crosses were all planted in 10×20 racks of tubes 1 inch wide and 8 inches deep. Every third row of 10 in each rack was left empty to allow for even air flow and light intensity and to reduce edge effects. The soil used was a 1:1 mixture of black soil and SunGro potting soil, blended with two teaspoons per square foot of Oscmocote Plus fertilizer. Plants were grown in growth chambers for 14 d (25° during 16-hr days and 20° during the nights).

The 27 NAM parental lines (Table S1) (Yu *et al.* 2008) were grown in three replications of 10 plants per line, with lines randomly distributed throughout the growth chamber. All healthy plants were sampled for histology, and 10 to 27 images were measured per line.

For the NAM subpopulations B73 × CML277 (Z005) and B73 × P39 (Z024), 140 and 137 lines, respectively, were examined (Table S1). These lines were grown in five replications of one plant per line, all in one chamber, with lines randomly distributed. In addition to the 140 and 137 RILs, all parental lines (B73, Mo17, P39, CML277) as well as 10 representative inbred lines (Table S1) from the IBM RIL population (selected to encompass the ranges of observed SAM height and SNP diversity) were included in each set for data standardization with the IBM RIL analyses in Thompson *et al.* 2014. Based on previous experiments, row–column variation for SAM architecture traits is trivial when compared with plant-to-plant and chamber-to-chamber variation, hence the decision to use five full replications of each genotype with adequate checks. All healthy plants were sampled for histology, and 1 to 5 images (average 3.8) were measured per line for RILs, 6 to 10 (average 8.3) were measured for each of the IBM controls, and 14 to 19 (average 16) were measured per parental line.

Eight NAM founders were crossed to both B73 and Mo17, and these 16 F1 crosses (Table S1) as well as the 10 parental inbreds were grown in 20 replications of one plant per replication, scattered randomly throughout each replication. All healthy plants were sampled, and 14 to 18 images were measured per line, with the exception of Hp301 and its crosses. These lines had very poor germination, and only nine, three, and one plants were measured for Hp301, Hp301 × B73, and Hp301 × Mo17, respectively.

The time course experiment included 18 lines, with five plants per line per time point. These were grown in the greenhouse in St. Paul, Minnesota in April/May 2014 in 6-inch square pots, 5 plants per pot, with soil mixture as described above. Greenhouse growth settings were 18-hr days, 27° during the days, and 21° during the nights.

### Histology

All samples were dissected and fixed in FAA and then processed according to one of the two methods described in Thompson *et al.* (2014): paraffin embedding/microtome sectioning or methyl salicylate tissue-clearing. The 27 NAM founders were embedded in paraffin and sectioned. All other experiments utilized the methyl salicylate tissue-clearing protocol.

### SAM architecture measurements

**Figure 1:**
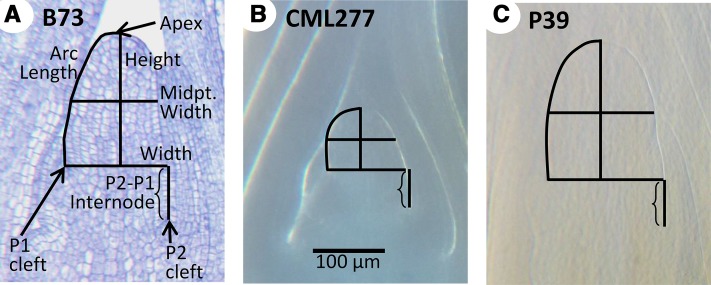
SAM phenotypes examined. Median longitudinal sections of the SAM in inbred lines B73 (A), CML277 (B), and P39 (C), indicating measurements taken (A). Compared to B73, P39 is similar in height but wider, with a height:width ratio close to 1 (*vs.* 1.26 in B73). CML277 is dramatically shorter than B73 and only slightly less wide, resulting in meristems typically shorter than their width (average ratio, 0.71). Images were obtained via histological section (A) or methyl salicylate clearing (B, C) but are all the same scale (B).

SAM images were measured using ImageJ software (Rasband 2014; http://rsbweb.nih.gov/ij/) according to Thompson *et al.* (2014). Briefly, SAM width, height, arc length, midpoint width, and plastochron internode length were measured relative to the P1 cleft (Figure 1A). The derived traits of height:width ratio and volume as a dome were also calculated for each sample. For the NAM founders, cells in the L1 layer along the arc length were counted and the derived trait of average cell size (arc length divided by arc cell number) was calculated; these two traits were not examined in the other datasets due to an inability to reliably see cell wall boundaries in images from cleared tissue.

### Data analysis

Raw phenotypic data analysis and visualization, linear models to standardize datasets, ANOVAs, Tukey’s HSD, and Pearson’s phenotypic correlations were all conducted in R (R Core Team 2014; http://www.r-project.org/). Entry mean-basis heritability was calculated based on the work of Bernardo (2010) as additive genetic variance of the RIL means [Va_RIL_=(MS_genotype_−MS_residual_)/reps] divided by total phenotypic variation among line means (Vp=Va_RIL_+MS_residual_).

QTL mapping was performed in QTLCartographer (Basten 1994, 2002) with genetic maps via Panzea (Buckler *et al.* 2009; http://www.panzea.org), using composite interval mapping with 10 background markers and a window size of 5 cM. Significance thresholds were determined via 1000 permutations at α=0.05 on each trait, and confidence intervals were calculated using a 1-LOD drop. Model proportion of variation explained was calculated using a linear model and ANOVA in R (R Core Team 2014; http://www.r-project.org/) to estimate the combined effect of QTL in the population using adjusted model R^2^.

Phenotypic correlations of SAM architecture with adult plant traits were conducted using Pearson’s correlation coefficient. Adult plant trait measurements for the NAM subpopulations were obtained from Panzea (raw NAM public phenotype data, 2006–2009, from http://www.panzea.org; see supplementary data). Raw trait values were averaged across environments (between 5 and 10 for each trait), and phenotypic correlations with SAM traits were required to be significant (*P* < 0.05) in at least half of the environments as well as the overall average to be reported. Details on adult plant trait phenotyping (collection methods and locations) can be found on Panzea (http://www.panzea.org).

### Data access

All raw data as well as means used for analyses can be found in the maize SAM diversity raw data and means spreadsheet (Table S5, Table S6, Table S7, Table S8, Table S9, Table S10, Table S11, Table S12, Table S13, and Table S14).

## Results

### Natural diversity in maize SAM architecture

To examine the extent of natural variation for SAM architecture, we examined 27 diverse inbred maize lines used as parents in the maize NAM population (Yu *et al.* 2008). The NAM parents (Table S1) were selected to represent both allelic and phenotypic diversity in maize. At least five plants were measured from at least three replicates of each genotype. Nine traits were measured to characterize SAM architecture: SAM height, arc length (the distance along the surface of the SAM from the apex to the P1 cleft), width, and midpoint width (width at the midpoint of SAM height) from the P1 cleft; cell number in the L1 layer along the arc length; plastochron internode difference (vertical distance between P1 and P2 clefts); and the derived traits of cell size in the L1 layer, height:width ratio, and volume (Figure 1A). The measurements of SAM traits in the NAM parents were used to estimate the phenotypic variation in maize (Table 1 and Table S2).

**Table 1:** SAM architecture trait summaries in NAM founders and two RIL subpopulations

NAM Founders (and Independent Measures of B73, Mo17, CML277, and P39)						
	B73/Mo17		NAM Founders			
Meristem trait*^c^*	B73	Mo17	Mean	Range*^a^*	*P**^b^*	Heritability
*Height (μm)	178	114	122	90–179	<0.001	0.84
*Arc length (μm)	208	152	155	119–217	<0.001	0.82
Width (μm)	142	158	140	116–169	<0.001	0.82
Midpoint width (μm)	117	131	116	99–138	<0.001	0.76
Volume (million μm^3^)	4.55	1.99	2.13	0.94–4.96	<0.001	0.83
*Height:width ratio	1.25	0.72	0.87	0.70–1.25	<0.001	0.89
Plastochron internode (μm)	77	73	63	47–79	0.0019	0.35
Cell count in arc	21.9	15.7	16.9	13.3–23.7	<0.001	0.55
Arc cell size (μm)	9.4	9.4	9.2	8.2–10.4	0.022	0.24
Z005 (B73 × CML277) population and parents						
	Parents		RILs			
Meristem trait	B73	CML277	Mean	Range*^a^*	*P**^b^*	Heritability
*Height (μm)	182	90	113	66–170	<0.0001	0.38
*Arc length (μm)	212	120	144	97–206	<0.0001	0.35
*Width (μm)	147	126	134	103–169	<0.0001	0.32
*Midpoint width (μm)	122	107	113	90–150	<0.0001	0.37
*Volume (million μm^3^)	5	10	2	5–4.3	<0.0001	0.33
*Height:width ratio	1.26	0.71	0.84	0.55–1.17	<0.0001	0.51
*Plastochron internode (μm)	67	55	65	43–93	0.0156	0.07
Z024 (B73 × P39) population and parents						
	Parents		RILs			
Meristem trait	B73	P39	Mean	Range^a^	*P*^b^	Heritability
Height (μm)	182	173	164	93–225	<0.0001	0.44
Arc length (μm)	212	211	200	122–265	<0.0001	0.44
*Width (μm)	147	165	156	124–192	<0.0001	0.42
*Midpoint width (μm)	122	145	132	103–169	<0.0001	0.53
Volume (million μm^3^)	5.01	4.68	4.24	1.1–8.6	<0.0001	0.39
*Height:width ratio	1.26	1.04	1.05	0.67–1.53	<0.0001	0.56
Plastochron internode (μm)	67	76	73	50–73	0.0246	0.06

a Range of line means for each trait.

b Significance of genotype in linear model.

c Asterisk (*) indicates significance of Tukey’s HSD between parents at *P* < 0.05.

**Figure 2:**
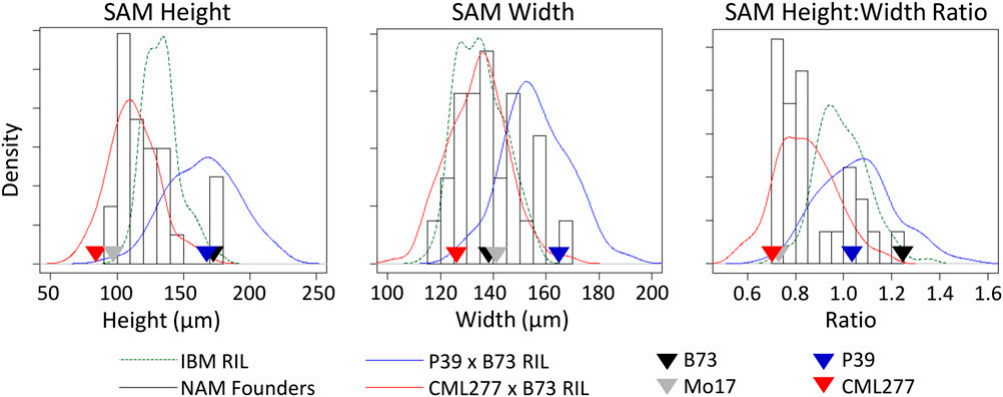
Distribution of main SAM traits across populations. Four inbred parental lines and distributions for their populations are shown for three traits. Although B73 and P39 are both very tall, P39 is much wider, leading to distinct ratios of SAM height:width. Despite their extreme values for SAM height and width relative to the other NAM founders, populations created from P39 and CML277 still showed transgressive segregation for all SAM traits when crossed to B73. Note: y-axis values differ for histograms and density distributions.

Distribution of the traits across NAM founder lines was approximately normal with the exception of SAM height:width ratio and one to three high outliers for most traits (Figure 2 and Figure S1, NAM founder histogram in black; Table S2). For SAM height, arc length, and volume, the inbred lines P39, B73, and Mo18W were atypically large, whereas IL14H was the outlier for SAM width (far right bars in black histograms, Figure 2). The ratio of SAM height to width showed a bimodal distribution, with approximately half of the lines centered around a mean of 0.75, half centered around 1, and a high outlier of 1.25 for B73. Average L1 cell size and plastochron internode difference showed lower heritabilities in the NAM founders and were likely more influenced by the environment than the other traits (Table 1).

### Heterosis for SAM architecture

**Figure 3:**
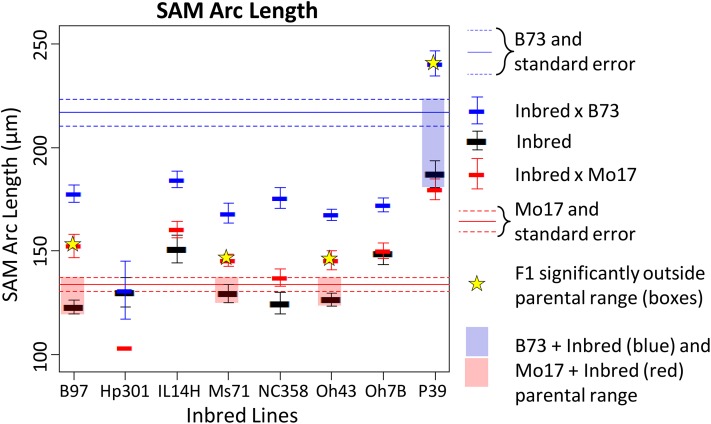
Presence of heterosis in NAM founder × B73/Mo17 crosses. Each of eight inbred NAM founder lines were crossed to B73 and Mo17 and examined for high-parent heterosis. Significant cases are shown starred and highlighted in blue (for crosses to B73) or red (for crosses to Mo17). Many lines showed heterosis when crossed to Mo17, for most traits. Only P39 exhibited heterosis when crossed to B73, indicating they contain unique alleles contributing to their large SAM size. Most inbred × B73 crosses showed near-midparent values.

Maize inbred lines B73 and Mo17 were crossed to eight diverse inbreds, and the resulting F1 progeny were examined for SAM architecture traits alongside the parental inbreds. Heterosis, defined here as SAM trait values significantly outside the range of the parents, was observed in three crosses made to Mo17 and one cross to B73 (Figure 3) for SAM arc length. Crosses exhibiting heterosis tended to be significant or near-significant across SAM traits (Figure S2). Some inbred lines, including P39 and Oh7B, appeared dominant to Mo17; SAM height, arc length, and height:width ratio in the Mo17-cross F1 were significantly different from Mo17 but equivalent to trait values for the other parental inbred. For B73, only crosses made to P39 showed values outside of parental ranges; SAM architecture of all remaining crosses to B73 appeared very near to mid-parent values.

Inbred background type was not a predictor of presence or strength of heterosis, because the non-stiff stalk lines B97 and Oh43 showed some of the highest levels of heterosis with Mo17 (also a non-stiff stalk) but seemed additive with B73 (stiff stalk). P39 and Il14H, although both sweet corn lines, exhibited very different responses when combined with B73 and Mo17; P39 was heterotic for SAM traits with B73, whereas Il14H was not significantly outside any parental ranges. SAM height and height:width ratio showed results similar to SAM arc length (Figure S2), whereas SAM width and midpoint width showed greatly reduced or effects, with only one cross (B97 × Mo17) exhibiting heterosis for SAM width in the F1.

Reciprocal crosses of B73 and Mo17 were also examined for SAM phenotypes. B73 × Mo17 and Mo17 × B73 both showed midparent values for all SAM architecture traits (example for SAM height shown in Figure S3). This result indicated a lack of parental effects and demonstrated that absence of SAM heterosis does not impact plant height or yield heterosis known to be exhibited by the F1 hybrids.

### Correlation of SAM architecture and growth with adult plant traits

**Figure 4:**
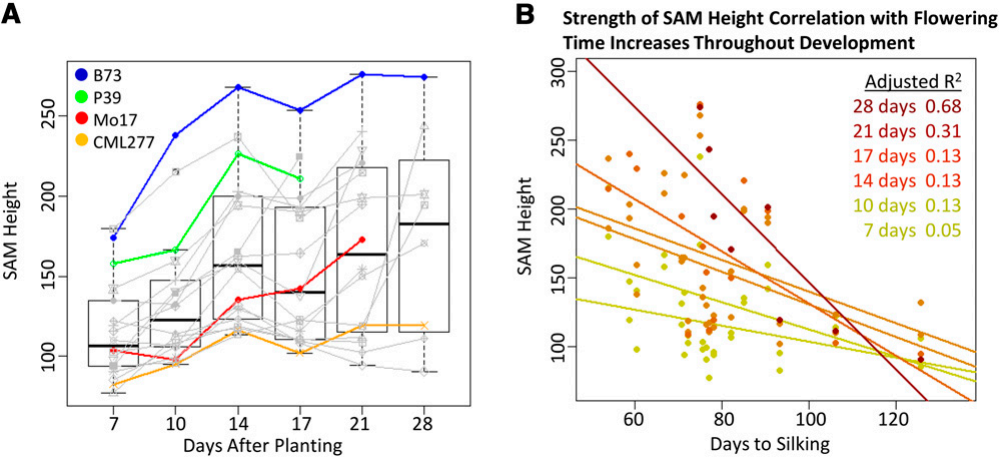
Time course of SAM growth in diverse lines and relationship of SAM height and flowering time. (A) Eighteen diverse genotypes (time point line means in colored and gray points/lines) sampled at six time points support a combined trend (box plots) of growth during vegetative development. Parental lines of the populations examined are shown in color. (B) Strength of correlation of SAM height and days to silking increases as the SAM approaches transition. Points shown are line means, and sloped lines are modeled for each time point.

To characterize the growth curve of the SAM throughout vegetative development and pinpoint the timing of transition relative to flowering time and SAM size, 18 inbred lines with diverse SAM architecture and flowering time were examined over a 4-wk period. Diverse SAM sizes were chosen based on preliminary data (M. Scanlon, unpublished results), and flowering time data were obtained from public resources (Hung *et al.* 2012; http://www.panzea.org). The resulting growth curve supported previous results describing three main phases of vegetative SAM growth (Thompson *et al.* 2014) (Figure 4A). In addition, correlation of SAM size with flowering time became significant approximately 3 wk after planting and increased throughout vegetative development; SAM height and days to silking are shown as an example in Figure 4B, but this relationship was true of SAM width and other flowering time measures as well.

**Figure 5:**
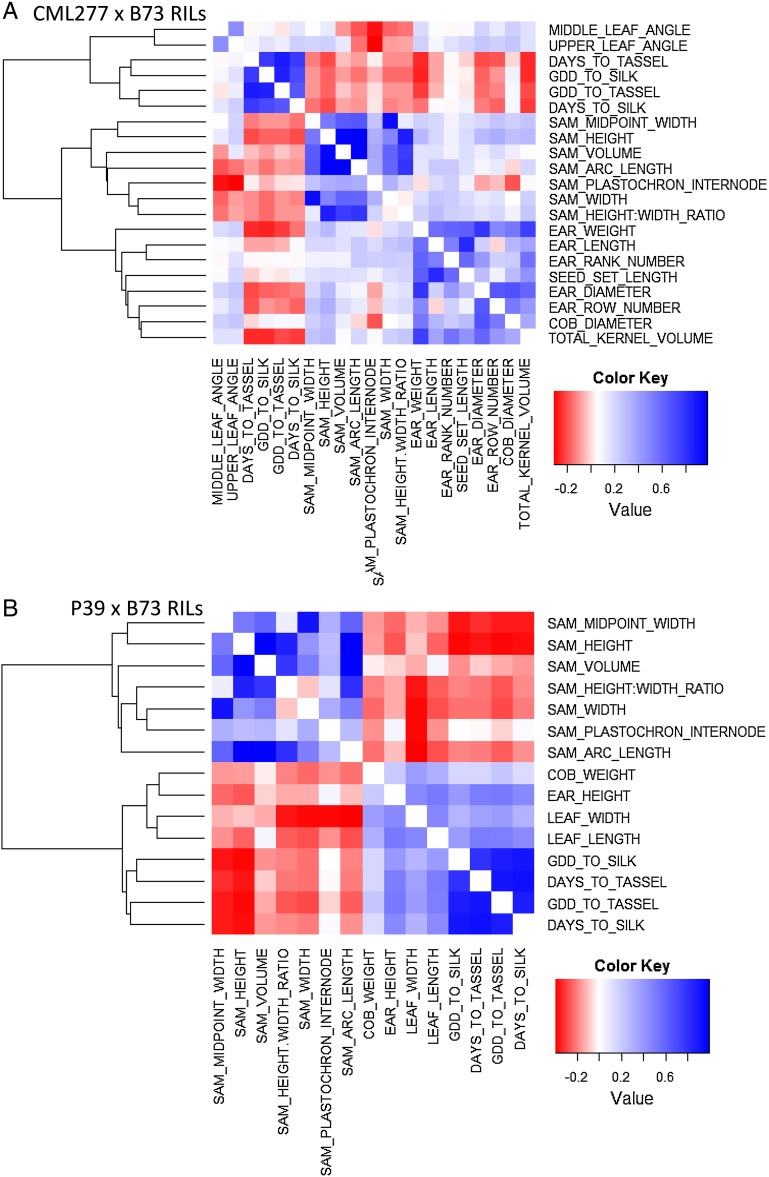
Correlations among SAM and adult plant traits. Adult plant traits significantly correlated with one or more of 7 SAM traits. Flowering time was related to SAM architecture in both populations. In the CML277 population (A), SAM width correlated with some yield measures, whereas in the P39 population (B) SAM height was related to leaf traits. All correlation values were moderate to weak (R2 ∼0.3), but robust, as correlations were required to be significant for the adult plant trait mean score as well as at least half of the individual environments.

To further investigate the relationship of SAM architecture with adult characteristics, public phenotype data from the NAM RILs (Hung *et al.* 2012; http://www.panzea.org) were leveraged to examine phenotypic correlations with SAM architecture (Figure 5, Table S3) in the B73 × P39 and B73 × CML277 NAM RIL subpopulations. These two populations were selected to encompass the range of phenotypic variation for both SAM height and width observed in the NAM founders, because P39 is very tall and wide, whereas CML277 is narrow and very short (Figure 1, B and C). The adult plant traits included several measures of flowering time, as well as tassel morphologies, plant and ear height, leaf length and width, leaf number and angle, cob diameter and weight, ear diameter and length, and several measures of yield (see full list of traits in Table S3). Pearson’s phenotypic correlations were used to identify traits significantly associated in a consistent manner across multiple environments with SAM architecture (see *Materials and Methods*). Briefly, correlations were required to be present in at least half of the field environments measured (between 5 and 10 for each trait) as well as for the overall trait mean across grow-outs to avoid false positives. Correlations observed were not strong (r ∼ 0.2–0.4), but they were significant (see Table S3). Associations with flowering time, leaf angle, leaf length and width, ear height, and cob weight were all negative; in other words, taller and/or wider meristems generally led to faster flowering, shorter and more narrow and upright leaves, lower ears, and lighter cobs. However, correlations with yield traits (ear diameter and length, seed set length, ear row and rank number, kernel volume, ear weight and diameter) were all positive, meaning that taller and/or wider meristems led to higher yield characteristics. Correlations with flowering time traits were present in both populations.

### QTL mapping in subpopulations derived from extreme founders

NAM RIL subpopulations derived from crosses of B73 to CML277 and P39 were examined for seven SAM traits: height, width, arc length, and midpoint width from the P1 cleft; plastochron internode distance; and the derived traits of height:width ratio and volume. In addition to extreme parental values for height and width, these subpopulations represented crosses between both similar and dissimilar parents in QTL mapping for certain traits, because both P39 and B73 are very tall but CML277 is very short. As expected, the combined resulting trait distributions encompassed the diversity present in the NAM founders (Figure 2 and Figure S1). Interestingly, although previous work identified B73 as exhibiting the maximum height, volume, and height:width ratio for maize SAM size in a biparental RIL population (Figure 2, IBM RIL distribution shown in dashed green line), RILs in the B73 × P39 population showed transgressive segregation for these traits (Figure 2, blue distribution line). However, in the RILs derived from B73 × CML277 (Figure 2, red distribution line), B73 was the highest value for these three traits, although transgressive segregation on the low end was observed.

**Figure 6:**
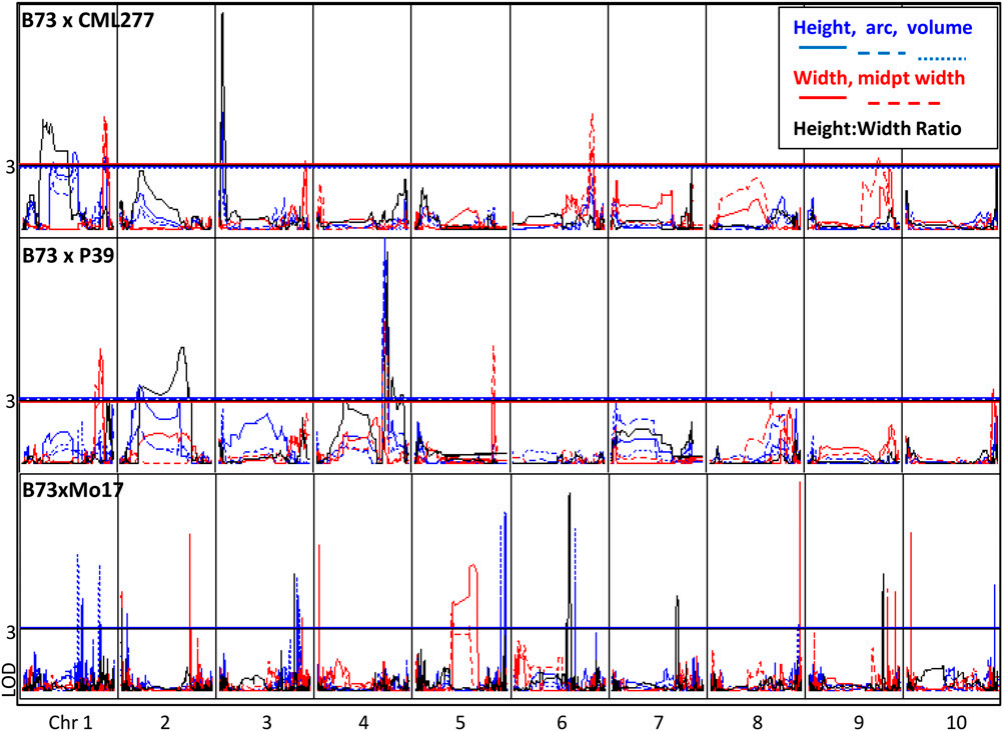
Comparison of quantitative trait loci associated with shoot apical meristem morphology in three populations. QTL across 10 chromosomes in two new populations as well as previously mapped IBMRIL for six traits. Height, arc length, and volume are shown in blue; width and midpoint width are shown in red; ratio is shown in black. QTL are not coincident across populations. In contrast to previous studies, some QTL are significant for both height and width traits.

For each of the seven SAM traits, one to five QTL were mapped per population. Estimated parental variance explained ranged from 6% to as high as 28%, with an average of 12% (Table 2). The 34 total QTL were distributed across nine chromosomes (all but chromosome 7). QTL regions for SAM architecture traits within a population frequently overlapped, even among low-correlated traits, for example, SAM height and width (Figure 6). Considering this co-localization of the 34 QTL, a total of 15 unique loci were identified, only one of which was identified in both of the populations (Table S4).

**Table 2:** Summary of SAM trait QTL

	# of QTL (+)*^a^*		Range of PVE*^b^*		Effect Range*^c^*		Model PVE*^d^*	
Meristem trait	Z005*^e^*	Z024*^f^*	Z005	Z024	Z005	Z024	Z005	Z024
Height (μm)	3 (3)	2 (1)	8–14%	8–25%	5.5–7.2	7.9–14.3	0.43	0.25
Arc length (μm)	2 (2)	1 (0)	7–13%	28%	5.4–7.1	15.9	0.28	0.28
Width (μm)	3 (3)	3 (0)	8–11%	8–13%	3.2–3.8	3.6–4.8	0.26	0.46
Midpoint width (μm)	3 (3)	5 (0)	8–12%	7–13%	2.8–3.5	3.3–4.8	0.34	0.56
Volume (million μm^3^)	3 (3)	1 (0)	7–12%	23%	1.7–2.3	7.9	0.37	0.27
Height:width ratio	2 (2)	4 (3)	12–25%	6–26%	0.044–0.065	0.043–0.086	0.39	0.39
Plastochron internode	1(1)	1(0)	14%	6%	3.5	2.6	0.18	0.07

a Numbers in parenthesis indicate QTL with positive effect in B73.

b Proportion of variation explained.

c Effect ranges shown are absolute values.

d Model proportion of variation explained; see *Materials and Methods* for details.

e Z005: B73 × CML277 RIL population.

f Z024: B73 × P39 RIL population.

## Discussion

### Extreme SAM architecture can exhibit transgressive segregation and heterosis

Previously reported data for the IBM RIL population (Thompson *et al.* 2014) indicated that the height, arc length, and volume of the maize SAM did not exceed that of B73. Even in the diverse NAM founder inbreds, B73 was one of the three tallest outliers for SAM height (Table 1 and Table S2, Figure 2). However, SAM measurements of a RIL population derived from B73 × P39 (another tall outlier) revealed transgressive segregation, leading to even larger SAM size (Table 1, Figure 2, and Table S1). Similarly, the RIL population derived from B73 × CML277 (the shortest of the NAM founders) exhibited a range of SAM sizes that included RILs shorter (or taller) than either of the two parents (Table 1, Figure 2, and Figure S1). These results indicate that the genes contributing to SAM size in these inbreds may be distinct, such that the additive effect or interaction among the new combinations of alleles leads to exceptional phenotypes.

Crosses of several NAM founders to B73 and Mo17 were also examined for SAM architecture to determine the presence of heterosis. Many crosses between NAM founder inbreds and Mo17 exhibited heterosis for SAM height, arc length, and height:width ratio, but they did not show significant differences from additive effects in SAM width and midpoint width. This indicates that unique SAM traits are inherently different in their levels of heterosis (Figure S2), and that heterosis for one trait is not predictive of that for unrelated traits. In addition, inbreds showed differences in heterosis when crossed. For example, although Mo17 hybrids were frequently larger than their parents, only one hybrid generated with inbred B73 was significantly larger than either parent. This cross that exhibited heterosis was the F1 generated by crossing the tall SAM of B73 and the tall and wide SAM of the early flowering sweet corn line P39. All other crosses to B73 showed F1 measures at or near midparent values (Figure 3, Figure S2), indicating that alleles for SAM architecture traits in B73 may frequently act in an additive fashion. P39 may contain unique alleles that lead to a larger meristem via additive effects or allelic interactions with B73.

Inbred P39 also showed a number of other unexpected results. In addition to exhibiting heterosis when crossed to B73, P39 seemed to have a dominant effect when crossed to Mo17, because P39 × Mo17 F1 values for SAM height, arc length, and height:width ratio were all significantly different from Mo17 but similar to P39 (Figure S2). This occurs in Oh7B × Mo17 F1 as well, with Oh7B alleles appearing to be dominant for the same three traits. Interestingly, heterosis did not seem to be predictable by inbred type (tropical, popcorn, sweet corn, stiff-stalk *vs.* non-stiff, etc.).

Heterosis for yield was also not a predictor of SAM architecture. B73 and Mo17 exhibit extensive heterosis for yield, yet F1 crosses between them were at midparent values for SAM traits. Interestingly, we observed phenotypic correlations of meristem morphology with whole plant traits (including yield). However, yield associations with SAM traits were only observed in the population where the meristems were extremely different sizes and the non-B73 parent was tropical; perhaps background effects and linked loci led to increased evidence of correlated traits. In addition to a lack of heterosis, reciprocal crosses of B73 and Mo17 revealed a lack of parental effects in the determination of meristem size (Figure S3).

### SAM size and rate of growth coincide with flowering time, as well as leaf and yield traits

Larger SAM size corresponds to earlier flowering, and the strength of this correlation increases throughout vegetative development (Figure 4B). The growth curve of the vegetative SAM in 18 diverse inbreds also reflects this difference in timing to maturity with earlier flowering lines transitioning into the reproductive phase earlier (Figure 4A). Therefore, it seems that flowering time in maize is influenced quite early in vegetative development, and this fate is reflected to some extent in the size of the vegetative SAM.

A phenotypic correlation analysis across the two NAM RIL subpopulations revealed several instances in which diversity in SAM architecture coincided with differences in whole-plant morphology. These whole-plant traits included four measures of flowering time: days to silk or tassel and growing degree days to silk or tassel. All four of these traits showed significant correlation with one or more SAM traits in both of the RIL populations (Figure 5, Table S3). Several leaf morphology and yield-related measurements also showed correlation with SAM height and width, respectively, in a population-specific manner (Figure 5). A connection between the growth pattern and form of the meristem and whole-plant architectures such as branching pattern has been previously discussed (see Sussex and Kerk 2001 for review), but our study correlates the physical architecture of the SAM—an undifferentiated cell structure—to that of differentiated organs in the adult plant.

### Major loci controlling SAM architecture can be shared among distinct traits within a population but differ by genetic background

The parents of the RIL populations differ greatly for meristem size. CML277 is short and narrow, with a height:width ratio less than 1 (Figure 2). Mo17 is similar in height and ratio, but wider, as wide as B73, and average among the NAM founders. However, B73 is much taller and therefore has a uniquely high height:width ratio. P39 shares the extreme height of B73 but is also very wide, giving it a ratio close to 1. Given these seemingly disjointed relationships between different meristem traits, it seems that SAM height and width are under distinct genetic control; this corroborates previous work in the IBM RIL population, where QTL for distinct traits did not overlap (Thompson *et al.* 2014). However, major loci mapped in the two NAM RIL subpopulations frequently showed colocalization of QTL for unrelated traits (Figure 6). Perhaps these populations contain genes not segregating in the IBM RILs that confer large effects to multiple traits; alternatively, the large-effect QTL overlapping for multiple unrelated traits could be caused by distinct genes (underlying multiple QTL) located very close together. This question requires fine-mapping to answer.

The 34 QTL mapped in the two NAM RIL subpopulations (Table 2) were able to be condensed to approximately 15 loci (nearly identical QTL intervals; see Table S4), each containing QTL for one or many traits. Only one of the 15 loci mapped for SAM traits overlapped between the two NAM RIL subpopulations (Figure 6, Table S4), and this locus did not confer a major effect. In the CML277 population (Z005), the chromosome 1 QTL was responsible for 7.3–12% of the variation in the population for SAM height, arc, volume, width, and midpoint width; it was also associated with height:width ratio in the P39 population (Z024), explaining only 6.5% (Table S4). An overlapping QTL was identified in previous SAM architecture QTL mapping in the IBM RIL population (Thompson *et al.* 2014), where it was significant for SAM arc length and height (Figure 6, Table S4). Here, again, it was a low-effect QTL, explaining ∼5% of the variation in the population (Thompson *et al.* 2014).

Given the lack of significant overlap in QTL among populations, variation in SAM architecture appears to be conferred by different genes in different genetic backgrounds, likely due to different segregating alleles in each population. This is consistent with the observations of transgressive segregation in the RIL populations and heterosis in the F1 hybrids. Large-effect genes are particularly cross-dependent, because neither of the major loci (effect contribution above 20%) detected here was present in any other population. It could be that the smaller-effect loci shared between populations represent downstream genes in the same or related pathways that serve to fine-tune control of SAM size and function.

## Supplementary Material

Supporting Information
