# Supplementary material for: Diversity of Maize Shoot Apical Meristem Architecture and Its Relationship to Plant Morphology
Source: G3 (Bethesda). 2015 Mar 5;5(5):819–27. doi: 10.1534/g3.115.017541 (PMC4426368; doi:10.1534/g3.115.017541)
Supplement: Supporting Information [file supp_5_5_819__index.html]

Diversity of Maize Shoot Apical Meristem Architecture and Its Relationship to Plant Morphology — Supporting Information 

# Diversity of Maize Shoot Apical Meristem Architecture and Its Relationship to Plant Morphology

## Supporting Information for Thompson *et al.*, 2015

**Files in this Data Supplement:**

- Supporting Information - Figures S1-S3 and descriptions of Tables S1-S14 (PDF, 444 KB)
- Figure S1 - SAM phenotype distributions. (PDF, 128 KB)
- Figure S2 - F1 SAM size. (PDF, 131 KB)
- Figure S3 - SAM height in B73-Mo17 reciprocal crosses. (PDF, 115 KB)
- Table S1 - Lines used in experiments. (.xlsx, 13 KB)
- Table S2 - SAM architecture traits across NAM founders. (.xlsx, 10 KB)
- Table S3 - Correlation values for SAM traits with adult plant traits. (.xlsx, 12 KB)
- Table S4 - QTL across populations. (.xlsx, 12 KB)
- Table S5 - Raw SAM architecture data from NAM founder inbreds, from three different growouts ("block"). (.xlsx, 75 KB)
- Table S6 - Line means of SAM architecture traits for NAM founder inbreds. (.xlsx, 10 KB)
- Table S7 - Raw SAM architecture data from B73 x CML277 and B73 x P39 NAM RIL populations, with parental and control inbreds. (.xlsx, 68 KB)
- Table S8 - Line means of SAM architecture traits for IBM RIL, B73 x CML277 RIL, and B73 x P39 RIL. (.xlsx, 44 KB)
- Table S9 - Adult plant phenotypes taken from panzea, with raw data for available field locations/years as well as calculated average. Environ\_code locations and years are described on the Panzea web site. (.xlsx, 565 KB)
- Table S10 - Raw SAM architecture data across timepoints for parental inbreds B73, Mo17, P39. (.xlsx, 23 KB)
- Table S11 - Raw SAM architecture data across timepoints for 18 diverse inbred lines. (.xlsx, 36 KB)
- Table S12 - Line means by timepoint for all measured inbreds combined. (.xlsx, 14 KB)
- Table S13 - Raw SAM architecture measurements of NAM founders x B73/Mo17 and inbreds. (.xlsx, 33 KB)
- Table S14 - Line means for NAM x B73/Mo17 and inbreds. (.xlsx, 9 KB)
