# Supplementary material for: Diversity of Maize Shoot Apical Meristem Architecture and Its Relationship to Plant Morphology
Source: G3 (Bethesda). 2015 Mar 5;5(5):819–27. doi: 10.1534/g3.115.017541 (PMC4426368; doi:10.1534/g3.115.017541)
Supplement: Supporting Information [file supp_g3.115.017541_017541SI.pdf]

G3 · Genes | Genomes | Genetics

Diversity of maize shoot apical meristem architecture and its relationship to plant morphology

Addie M. Thompson[*], Jianming Yu[§], Marja C. P. Timmermans[†], Patrick Schnable[§], James C. Crants[*], Michael J. Scanlon[‡], and Gary J. Muehlbauer[*,**]

[*]Department of Agronomy and Plant Genetics, University of Minnesota, St. Paul, Minnesota 55108
[§]Department of Agronomy, Iowa State University, Ames, Iowa 50011.
[†]Cold Spring Harbor Laboratory, Cold Spring Harbor, New York 11724.
[‡]Department of Plant Biology, Cornell University, Ithaca, New York 14853.
[**]Department of Plant Biology, University of Minnesota, Saint Paul, Minnesota 55108.

Corresponding author:
Gary J. Muehlbauer
Plant Biology
Room 250 Biological Sciences
1445 Gortner Avenue
Saint Paul, MN 55108
612-624-2755
muehl003@umn.edu

DOI: 10.1534/g3.115.017541

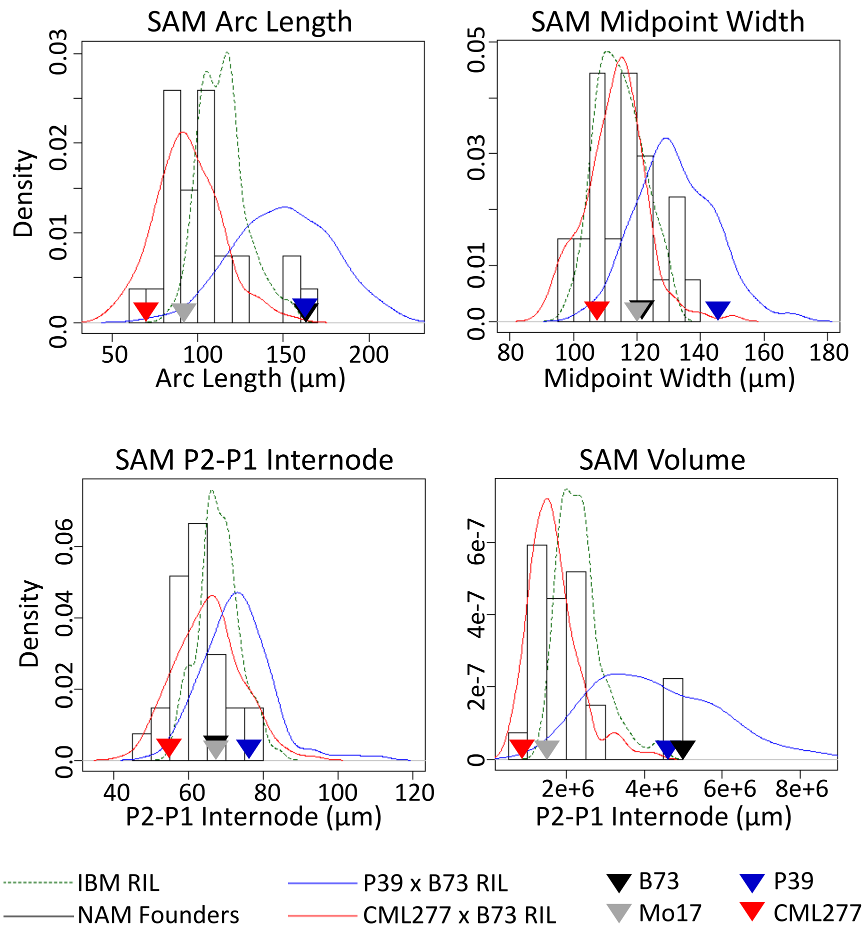

Figure S1. SAM phenotype distributions. Four inbred parental lines and distributions for their populations for remaining traits. SAM height, arc length, and volume show similar distributions; SAM width and midpoint width are highly similar as well. P2-P1 internode is most similar to SAM widths, while SAM heigh:width ratio captures largely unique information that is slightly related to SAM volume. These similarities reflect correlations observed among the traits. SAM width-related traits have very normal distributions, while height-related traits seem to be nearly bi-modal due to a small number of higher values.

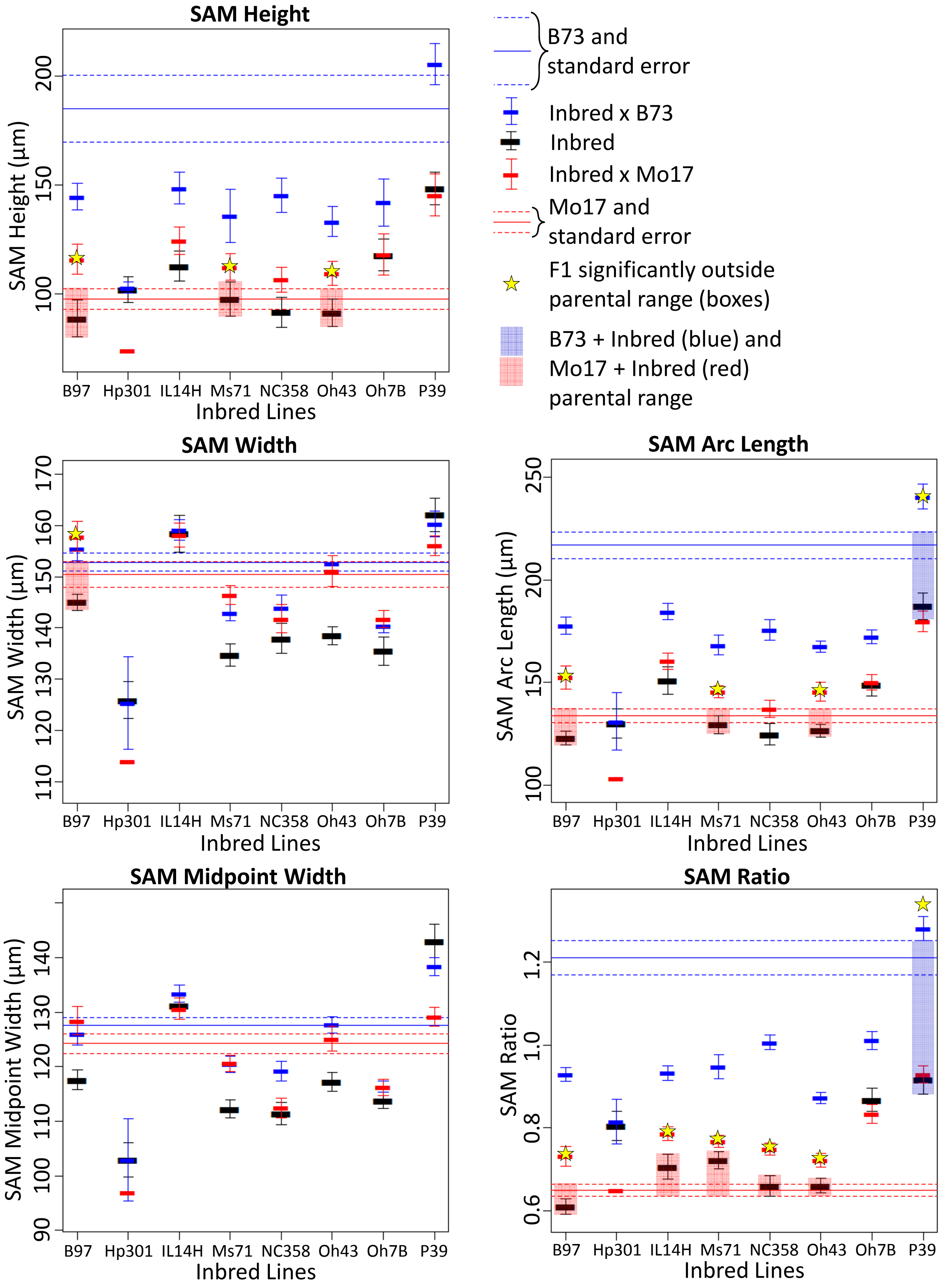

Figure S2. F1 SAM size. Each of eight inbred NAM founder lines were crossed to B73 and Mo17 and examined for outside-parent heterosis. Significant cases are shown starred and highlighted in blue (for crosses to B73) or red (for crosses to Mo17). Many lines showed heterosis when crossed to Mo17, for most traits. Only P39 exhibited heterosis when crossed to B73, indicating they contain unique alleles contributing to their large SAM size. Most inbred x B73 crosses showed near-midparent values.

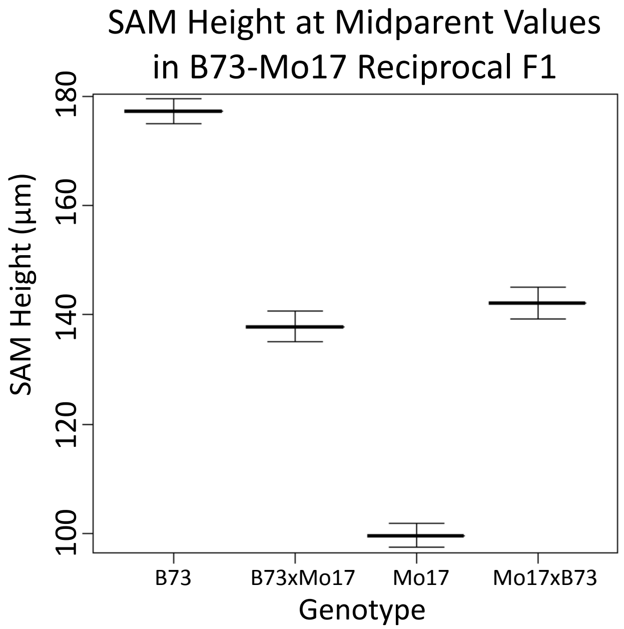

Figure S3. SAM height in B73-Mo17 reciprocal crosses. Reciprocal crosses between B73 and Mo17 show F1 SAM height near midparent values, indicating a lack of heterosis for meristem height in this cross and absence of parental effects.

Tables S1-S14

Available for download as Excel files at http://www.g3journal.org/lookup/suppl/doi:10.1534/g3.115.017541/-/DC1

Table S1. Lines used in experiments

Table S2. SAM architecture traits across NAM founders

Table S3. Correlation values for SAM traits with adult plant traits

Table S4. QTL across populations.

Table S5. Raw SAM architecture data from NAM founder inbreds, from three different growouts ("block")

Table S6. Line means of SAM architecture traits for NAM founder inbreds

Table S7. Raw SAM architecture data from B73 x CML277 and B73 x P39 NAM RIL populations, with parental and control inbreds

Table S8. Line means of SAM architecture traits for IBM RIL, B73 x CML277 RIL, and B73 x P39 RIL

Table S9. Adult plant phenotypes taken from panzea, with raw data for available field locations/years as well as calculated average. Environ_code locations and years are described on the Panzea web site.

Table S10. Raw SAM architecture data across timepoints for parental inbreds B73, Mo17, P39

Table S11. Raw SAM architecture data across timepoints for 18 diverse inbred lines

Table S12. Line means by timepoint for all measured inbreds combined

Table S13. Raw SAM architecture measurements of NAM founders x B73/Mo17 and inbreds

Table S14. Line means for NAM x B73/Mo17 and inbreds
