# Supplementary material for: Diversity of Maize Shoot Apical Meristem Architecture and Its Relationship to Plant Morphology
Source: G3 (Bethesda). 2015 Mar 5;5(5):819–27. doi: 10.1534/g3.115.017541 (PMC4426368; doi:10.1534/g3.115.017541)
Supplement: Supporting Information [file supp_g3.115.017541_FigureS1.pdf]

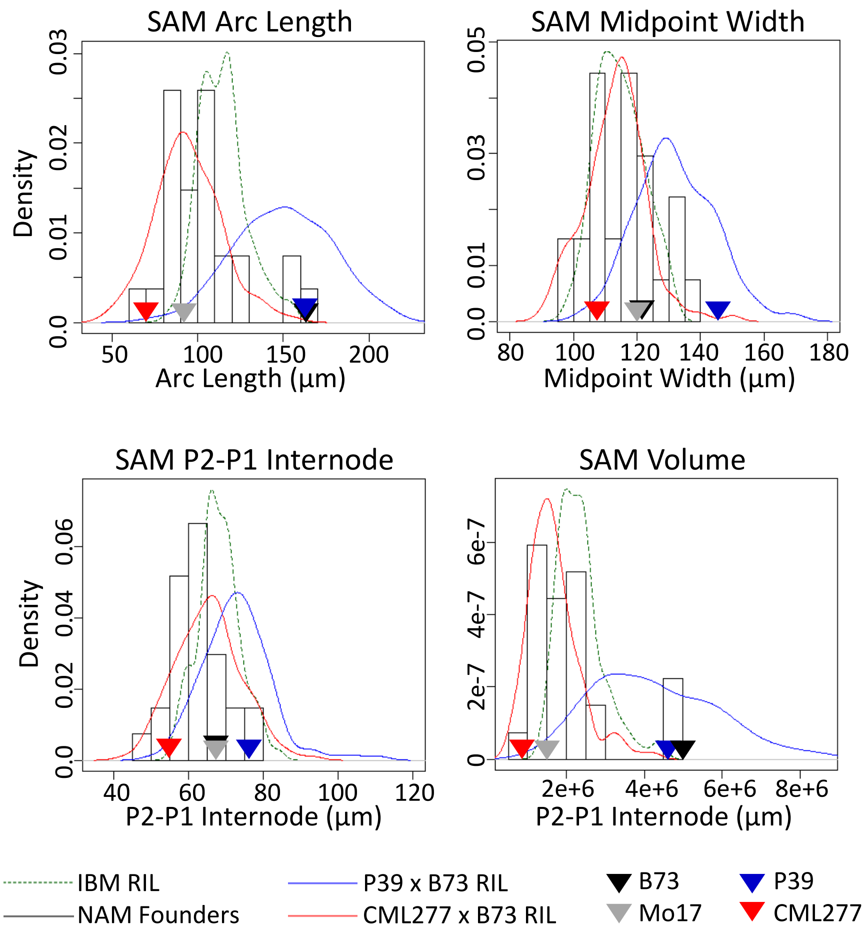

Figure S1. SAM phenotype distributions. Four inbred parental lines and distributions for their populations for remaining traits. SAM height, arc length, and volume show similar distributions; SAM width and midpoint width are highly similar as well. P2-P1 internode is most similar to SAM widths, while SAM heigh:width ratio captures largely unique information that is slightly related to SAM volume. These similarities reflect correlations observed among the traits. SAM width-related traits have very normal distributions, while height-related traits seem to be nearly bi-modal due to a small number of higher values.
