# Supplementary material for: Diversity of Maize Shoot Apical Meristem Architecture and Its Relationship to Plant Morphology
Source: G3 (Bethesda). 2015 Mar 5;5(5):819–27. doi: 10.1534/g3.115.017541 (PMC4426368; doi:10.1534/g3.115.017541)
Supplement: Supporting Information [file supp_g3.115.017541_FigureS2.pdf]

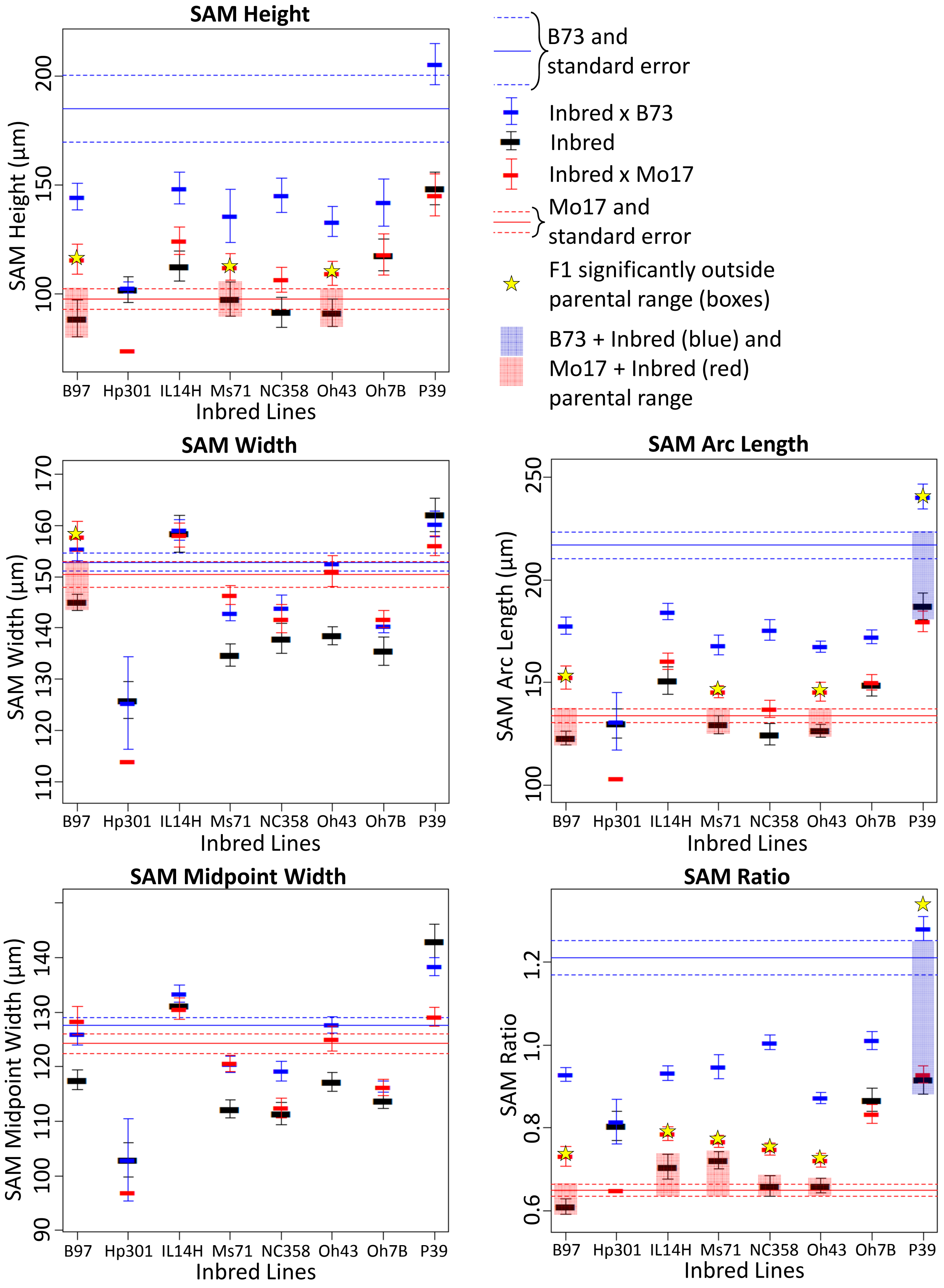

Figure S2. F1 SAM size. Each of eight inbred NAM founder lines were crossed to B73 and Mo17 and examined for outside-parent heterosis. Significant cases are shown starred and highlighted in blue (for crosses to B73) or red (for crosses to Mo17). Many lines showed heterosis when crossed to Mo17, for most traits. Only P39 exhibited heterosis when crossed to B73, indicating they contain unique alleles contributing to their large SAM size. Most inbred x B73 crosses showed near-midparent values.
