# Supplementary material for: Diversity of Maize Shoot Apical Meristem Architecture and Its Relationship to Plant Morphology
Source: G3 (Bethesda). 2015 Mar 5;5(5):819–27. doi: 10.1534/g3.115.017541 (PMC4426368; doi:10.1534/g3.115.017541)
Supplement: Supporting Information [file supp_g3.115.017541_FigureS3.pdf]

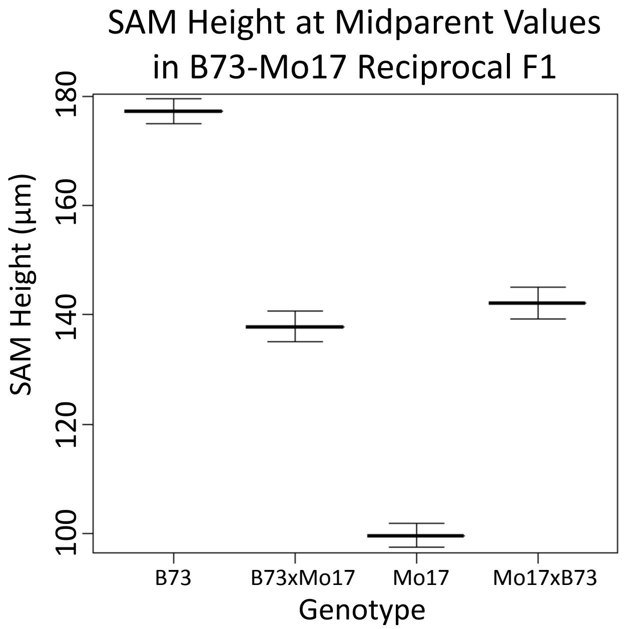

Figure S3. SAM height in B73-Mo17 reciprocal crosses. Reciprocal crosses between B73 and Mo17 show F1 SAM height near midparent values, indicating a lack of heterosis for meristem height in this cross and absence of parental effects.
